# miR-214 Protects Against Uric Acid-Induced Endothelial Cell Apoptosis

**DOI:** 10.3389/fmed.2020.00411

**Published:** 2020-08-05

**Authors:** Bingyu Yang, Shuzhen Li, Jun Zhu, Songming Huang, Aihua Zhang, Zhanjun Jia, Guixia Ding, Yue Zhang

**Affiliations:** ^1^Department of Nephrology, Children's Hospital of Nanjing Medical University, Nanjing, China; ^2^Jiangsu Key Laboratory of Pediatrics, Nanjing Medical University, Nanjing, China; ^3^Nanjing Key Laboratory of Pediatrics, Children's Hospital of Nanjing Medical University, Nanjing, China

**Keywords:** miR-214, uric acid, endothelial cells, COX-2, apoptosis

## Abstract

**Background:** Uric acid (UA) has been reported to be an important risk factor for cardiovascular diseases and can cause endothelial cell apoptosis through unclear mechanisms. Accumulating evidence has demonstrated that miR-214 plays a pivotal role in the pathogenesis of cardiovascular diseases. This study was to investigate the role of miR-214 in UA-induced endothelial cell apoptosis and the underlying mechanism.

**Material and methods:** We enrolled 30 patients with hyperuricemia and 32 healthy controls and analyzed the levels of miR-214 in the serum of the participants. Then mouse aorta endothelial cells (MAECs) were treated with UA to induce cell apoptosis. An miR-214 mimic and a specific COX-2 inhibitor (NS398) were used to confirm the roles of these molecules in mediating UA-induced MAEC apoptosis or COX-2/PGE_2_ cascade activation.

**Results:** A significant reduction in circulating miR-214 in the hyperuricemia patients compared with the healthy controls, along with a negative correlation with UA levels was observed. In the MAECs, UA treatment strikingly increased apoptosis as shown by the upregulation of BAX and cleaved Caspase-3 and the increased number of apoptotic cells. Interestingly, the expression of COX-2 was also upregulated at both the protein and mRNA levels during UA-induced cell apoptosis. In addition, an miR-214 mimic blocked UA-induced MAEC apoptosis, COX-2 induction and PGE_2_ secretion. The inhibition of COX-2 markedly ameliorated UA-induced apoptotic response and PGE_2_ production in MAECs. Luciferase activity assays further confirmed that COX-2 is a target gene of miR-214 in endothelial cells.

**Conclusion:** We concluded that miR-214 could alleviate UA-induced MAEC apoptosis possibly by inhibiting the COX-2/PGE_2_ cascade.

## Introduction

Uric acid (UA) is the final product of purine metabolism. Hyperuricemia is the main cause of gout. Increasing evidence showed that hyperuricemia is also closely associated with cardiovascular and renal diseases (1, 2). Vascular endothelial dysfunction leads to the development of many cardiovascular diseases (CVDs) and promotes the progression of CVDs (3). Recently, UA was found to cause vascular endothelial cell apoptosis *in vivo* (4). In clinic, hyperuricemia is also considered to be an independent risk factor for the development of hypertension and atherosclerosis (5–8), which triggered our interest to investigate the pathogenic mechanism of UA-induced endothelial cell injury.

microRNAs (miRNAs) are a kind of single-stranded non-coding small molecule RNA with a length of 17–22 nucleotides. They are mainly involved in the posttranscriptional regulation of gene expression and highly conserved in evolution (9). Increasing evidence revealed that miRNAs play key roles in the development of cardiovascular diseases (10–13). Recent studies have demonstrated that miR-214 is involved in the pathogenesis of cardiac fibrosis (14), myocardial injury (15), endothelial cell angiogenesis (16, 17), and inflammatory responses (18). Some studies have also shown that the dysregulation of miR-214 contributes to the pathogenesis of pulmonary hypertension (19, 20). In addition, miR-214 is known for its role in attenuating apoptosis (21). However, whether miR-214 plays a role in regulating UA-induced endothelial cell apoptosis is still unknown.

Cyclooxygenase-2 (COX-2) is an critical enzyme mediating the production of prostaglandins (PGs), including prostaglandin E_2_ (PGE_2_) in physiological and disease conditions (22, 23). A recent study indicated that UA increased COX-2 expression and PG synthesis (24). Another study showed that the use of a COX-2 inhibitor in mouse aortic endothelial cells significantly inhibited cell apoptosis and PGE_2_ secretion induced by PM2.5 (25). However, the role of the COX-2/PGE_2_ cascade in UA-induced endothelial cell injury has not been defined.

According to bioinformatics analysis, COX-2 is a potential target gene of miR-214. To elucidate the contribution of miR-214 to the pathogenesis of UA-induced apoptosis in endothelial cells, we observed the circulating levels of miR-214 in the patients with hyperuricemia and in endothelial cells challenged with UA, investigated the function of miR-214 and COX-2 in UA-induced endothelial apoptosis, and examined the regulation of miR-214 on COX-2 in the present study. The results demonstrated an important role of miR-214 in UA-induced apoptosis, possibly mediated by the direct targeting of COX-2.

## Materials and Methods

### Materials

Dulbecco's modified Eagle's medium (DMEM), trypsin solution (EDTA), fetal bovine serum (FBS) and penicillin-streptomycin were purchased from Gibco (Invitrogen, Grand Island, NY). UA was obtained from Sigma (St. Louis, MO). The mouse miR-214 mimic was provided by GenePharma Co., Ltd. (Shanghai, China). The COX-2 inhibitor NS-398 (catalog no. s1772) was purchased from Beyotime (Shanghai, China). The PGE_2_ enzyme immunoassay kit and the anti-COX-2 antibody were obtained from Cayman Chemicals (Ann Arbor, MI). The anti-BAX and GAPDH antibodies were provided by Proteintech (Rosemont, 90 USA). Anti-cleaved Caspase-3 antibody was purchased from Cell Signaling Technology (Danvers, MA).

### Patients

Blood from 30 hyperuricemia patients who were newly diagnosed in the Affiliated Hospital of Nanjing Medical University (Nanjing, China) and 32 healthy controls was collected for the analysis of serum miR-214 levels. Hyperuricemia was diagnosed according to UA concentration ≥417 μmol/L in males or ≥357 μmol/L in females (26). Clinical parameters, including gender, age, uric acid, urea nitrogen, glucose, triglycerides, and total cholesterol were collected (Table 1). All included hyperuricemia patients had no clinical features of gout and treatments of urate-lowering or anti-inflammation drugs. The healthy controls were subjects without abnormalities in physiological indexes including blood uric acid (Table 1). The protocol concerning the use of the patients' samples and the clinical data in this study was approved by the Human Subjects Committee of Nanjing Medical University. Informed consent was obtained from all participants. The serum was separated from the blood samples of 30 hyperuricemia patients and 32 normal controls by centrifugation at 2,000 g for 10 min and collected for the analysis of miR-214 expression.

**Table 1:** General data of the hyperuricemia patients and the controls.

**Group**	**Gender (*n* male/*n* female)**	**Age (years)**	**UA (μmol/L)**	**BUN (mmol/L)**	**Glu (mmol/L)**	**TG (mmol/L)**	**TCHO (mmol/L)**
Hyperuricemia (*N* = 30)	26/4	40.73 ± 2.07	615 ± 17.1^c^	8.37 ± 1.5^a^	5.84 ± 0.50	2.1 ± 0.28^b^	4.44 ± 0.22
Non-hyperuricemia (*N* = 32)	18/14	38.19 ± 1.72	290.2 ± 12.72	5.23 ± 0.2	5.72 ± 0.29	1.28 ± 0.15	4.62 ± 0.13

a *P < 0.05*;

b *P < 0.01*;

c *P < 0.0001 vs. Non-hyperuricemia*.

*UA, uric acid; BUN, blood urea nitrogen; Glu, glucose; TG, triglyceride; TCHO, total cholesterol*.

### Cell Culture and Oligonucleotide Transfection

The mouse aorta endothelial cells (MAECs), obtained from Jennio Biotech Co. Ltd. (Guangzhou, China), were cultured in DMEM medium containing 10% FBS and 1% streptomycin in a humidified 5% CO_2_ atmosphere incubator maintained at 37°C. The cells were digested with 0.25% trypsin (EDTA) and sub-cultured into 6- or 12-wells plates for 24 h. Cells were grown to 60–70% confluence and transfected with miR-214 mimic (40 nM) and negative control (40 nM) using a Lipofectamine 2,000 Kit (Invitrogen; Thermo Fisher Scientific, Inc.) according to the manufacturer's instructions. Then UA was added to the serum-free medium to stimulate MAECs for 24 h.

### Quantitative Real-Time PCR (qRT-PCR)

Total RNA was extracted using TRIzol (TaKaRa) according to the manufacturer's instructions. The relative expression of miR-214 was assessed by an Applied Biosystems 7,500 Sequence Detection system (Thermo Fisher Scientific, Inc.) using a SYBR PrimeScript miRNA RT-PCR kit (Takara Bio, Inc., Otsu, Japan); miR-214 expression was normalized to U6. Bulge-loopTM miRNA qRT-PCR Primer Sets specific for miR-214 were designed by RiboBio (Guangzhou, China). cDNAs were synthesized from 1 μg of total RNAs using the TaKaRa PrimeScript^TM^ RT Master Mix kit following the manufacturer's instructions. Real-time quantitative PCR of Caspase-3 and BAX was performed using an ABI 7,500 Sequence Detection system with a SYBR Green PCR Master Mix and normalized to GAPDH. The sequences of the primer genes are shown in Table 2. The PCR cycle consisted of an initial denaturing period at 95°C for 5 min, followed by 40 cycles of 95°C for 30 s, 60°C for 30 s, and 72°C for 30 s. The mRNA levels were calculated using the delta-delta Ct method.

**Table 2:** Primer sequences for qRT-PCR.

**Gene symbol**	**Primer sequences**
COX-2	5′-AGGACTCTGCTCACG AAGGA-3′
	5′-TGACATGGATTGGAACAGCA-3′
BAX	5′-CCGGCGAATTGGAGATGAACT-3′
	5′-CCAGCCCATGATGGTTCTGAT-3′
Caspase-3	5′-ATGGGAGCAAGTCAGTGGA-3′
	5′-GGCTTAGAATCACACACACAAAG-3′
GAPDH	5′-GTCTTCACTACCATGGAGAAGG-3′
	5′-TCATGGATGACCTTGGCCAG-3′

### Western Blotting

The MAECs were lysed in RIPA buffer containing the protease inhibitors at 4°C for 30 min, and the extracts were centrifuged at 12,000 rpm for 15 min at 4°C. The protein concentration was determined by a BCA Protein Assay Kit (Beyotime), then the samples were subjected to SDS-PAGE and transferred to PVDF membranes. The PVDF membranes were blocked with 5% non-fat milk for 1 h and incubated with primary antibodies against BAX (1:1000), cleaved Caspase-3 (1:1000), and COX-2 (1:500) overnight at 4°C. After washing, the membranes were incubated with HRP-tagged secondary antibody at room temperature for 1 h. The intensity of the target protein bands was measured using ImageJ software (NIH, Bethesda, MD, USA). Protein expression was normalized to GAPDH.

### Luciferase Reporter Assay

The target genes of miR-214 were predicted using TargetScan (http://www.targetscan.org/index.html) and miRanda (http://www.microrna.org). Plasmids containing the wild-type COX-2 3'-UTR (PmirGLO-COX-2 3'-UTR-WT) or a mutant COX-2 3'-UTR (PmirGLO-COX-2-3′-UTR-Mut) were obtained from GenePharma Co., Ltd. (Shanghai, China). MAECs were co-transfected with PmirGLO-COX-2-3′-UTR-WT or PmirGLO-COX-2-3′-UTR-Mut together with a miR-214 mimic or miR-NC. Renilla luciferase plasmid (pGL4.73, Promega) was used as luciferase activity control. After 48 h transfection, luciferase activity was measured with the Luciferase Reporter Assay System (Promega Corporation), following the manufacturer′s protocol.

### Apoptosis Assay

After treatment, the MAECs were collected in suspension and were washed with PBS. The cells were double-stained with FITC-Annexin V and propidium iodide (PI) according to the manufacturer's instructions. Quantification was performed by flow cytometry (Bedford, MA), and the data analysis was performed by FlowJo software.

### Enzyme Immunoassay (EIA)

The cell culture medium was collected after UA treatment with or without a pretreatment with miR-214 mimic or COX-2 inhibitor (NS-398), and the concentration of PGE_2_ was determined by an EIA kit (Cayman Chemical) following the manufacturer′s instructions.

### Statistical Analysis

Differences between the groups were statistically analyzed using ANOVA followed by Bonferroni's test or an unpaired Student's *t*-test. The degree of associations between variables were determined by Pearson correlation analysis. To rule out the impact of BUN and TG, the correlation coefficient between miR-214 and UA were determined by partial correlation analysis with BUN and TG adjustment, and multiple variable linear regression models after adjusting BUN and TG were used to further explore the associations between serum UA and miR-214 level, with the results presented as regression coefficients (beta) and 95% confidence intervals (CIs). We performed above data analyses using GraphPad Prism 6 software (GraphPad, San Diego, CA) and SPSS version 20.0 (SPSS, Inc., Chicago, IL). All data are presented as the mean and standard error (SE). *P* < 0.05 was considered statistically significant.

## Results

### miR-214 Was Lower in the Serum of Hyperuricemia Patients Compared With the Healthy Controls

**Figure 1:**
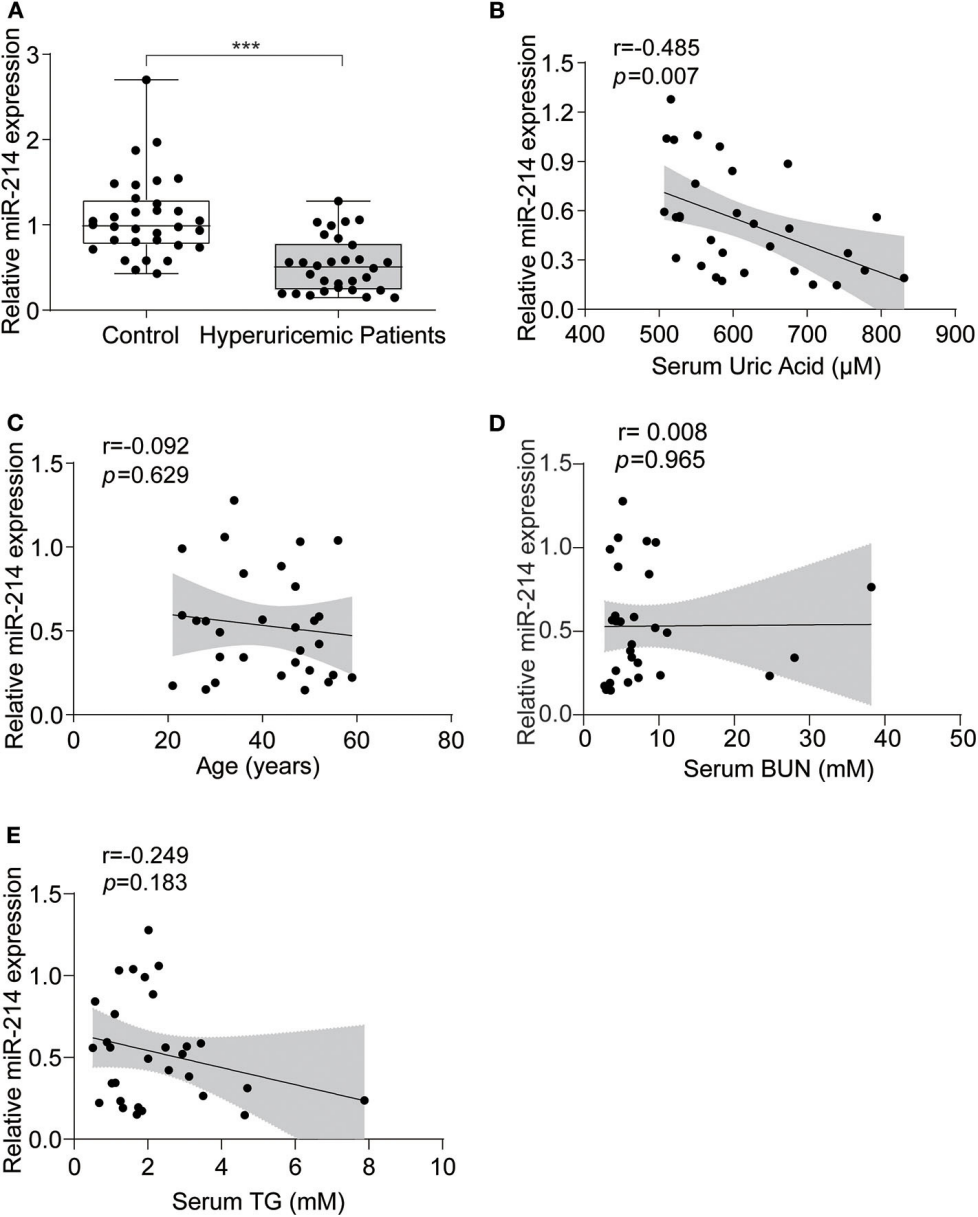
miR-214 expression was decreased in the serum of hyperuricemia patients. **(A)** Serum samples from 30 hyperuricemia patients and 32 age-and sex-matched healthy controls were collected, and the level of miR-214 expression in the serum was assessed by qRT-PCR. **(B)** Pearson correlation analysis between serum uric acid and serum miR-214 levels in hyperuricemia patients. **(C)** Pearson correlation analysis between age and serum miR-214 levels in hyperuricemia patients. **(D)** Pearson correlation analysis between BUN and serum miR-214 levels in hyperuricemia patients. **(E)** Pearson correlation analysis between TG and serum miR-214 levels in hyperuricemia patients. Values are means ± SE; *n* = 30 in the hyperuricemia group and *n* = 32 in the control group. ***P < 0.001.

To determine the expression of miR-214 in hyperuricemia patients, we measured the level of miR-214 in the sera of hyperuricemia patients and sex- and age-matched healthy controls. As shown in Figure 1A, the expression of miR-214 in the serum of the hyperuricemia patients was significantly lower than that in the healthy controls. Meanwhile, these hyperuricemia patients also showed higher levels of BUN and TG compared with healthy controls (Table 1). By Pearson correlation analysis, we found a negative correlation between circulating miR-214 and serum uric acid (Figure 1B) but not age, BUN and TG (Figures 1C–E). Furthermore, after adjusting for BUN and TG, we still observed a significant correlation between the circulating miR-214 and serum uric acid (coefficient = −0.002; 95% CI = −0.003, 0; *P* = 0.0365). These data suggested a potential role of the reduced circulating miR-214 in the vascular pathology of hyperuricemia patients.

### UA Reduced the Expression of miR-214 in MAECs

**Figure 2:**
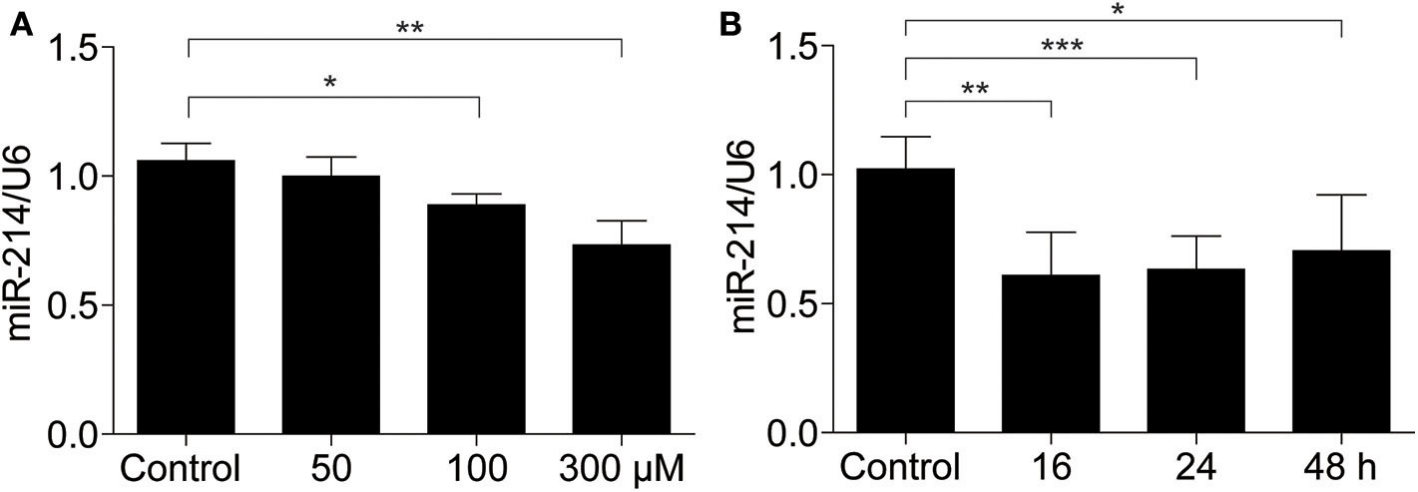
miR-214 was downregulated in murine aortic endothelial cells exposed to uric acid. **(A)** MAECs were treated with different doses of UA (0, 50, 100, and 300 μM) for 24 h, and then miR-214 expression was assessed by qRT-PCR. **(B)** miR-214 expression level in MAECs after 300 μM UA exposure for 0, 16, 24, and 48 h was determined by qRT-PCR. U6 was used as the internal control. All values are means ± SE; *n* = 3 in each group. *P < 0.05, **P < 0.01, ***P < 0.001.

Next, using qRT-PCR, we examined miR-214 expression in MAECs following UA stimulation. Our results demonstrated that the miR-214 mRNA levels were decreased in a dose-dependent manner after UA treatment (0, 50, 100, and 300 μM) compared with the control group (Figure 2A). Furthermore, we treated the endothelial cells with UA (300 μM) at different time points (0, 16, 24, and 48 h). As shown in Figure 2B, UA downregulated miR-214 mRNA expression in a time-dependent manner.

### UA Significantly Enhanced Apoptosis in MAECs

**Figure 3:**
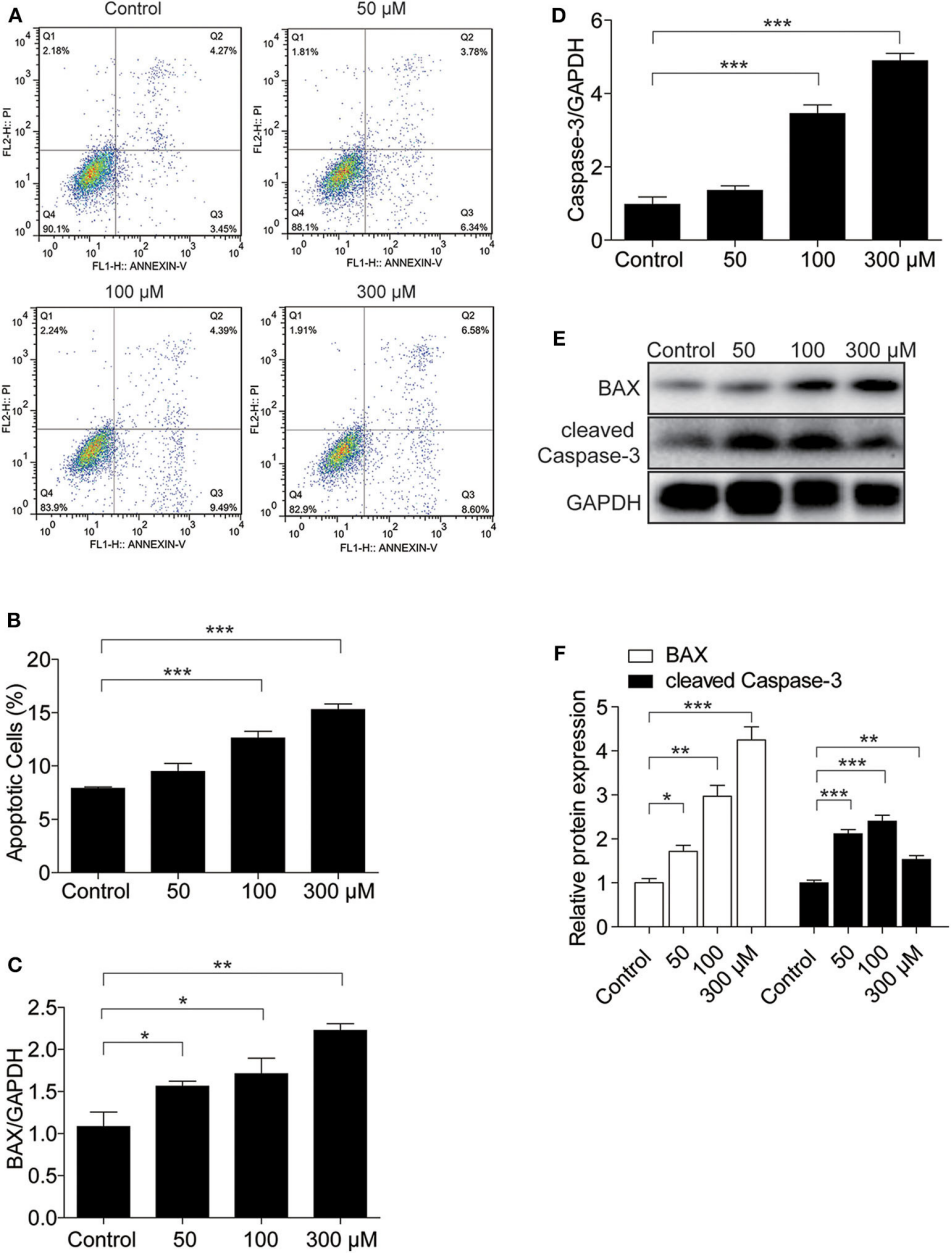
UA treatment led to MAEC apoptosis. MAECs were treated with the indicated doses of UA for 24 h. **(A)** Flow cytometry was used to determine the percentages of Annexin V-FITC+ cells (right lower quadrant, early apoptosis) and Annexin V-FITC+/PI+ cells (right upper quadrant, late apoptosis), and the representative images of flow cytometry were presented. **(B)** The percentage of apoptotic cells was analyzed. **(C,D)** mRNA levels of BAX and Caspase-3 were analyzed by qRT-PCR. **(E)** Protein levels of BAX and cleaved Caspase-3 were assessed by Western blotting. **(F)** Quantification of the Western blots of BAX and cleaved Caspase-3 in **(F)**. GAPDH was used as an internal control. All values are means ± SE; *n* = 3 in each group. *P < 0.05, **P < 0.01, ***P < 0.001.

To investigate whether UA could induce endothelial cell apoptosis, we treated endothelial cells with different doses of UA at different time points. First, we used flow cytometry to analyze cell apoptosis in endothelial cells exposed to different doses of UA. UA was found to induce a significant increase in the number of apoptotic cells (Figures 3A,B). In addition, BAX and Caspase-3 expression were elevated at the mRNA level by UA in a dose-dependent manner, as detected by qRT-PCR (Figures 3C,D). We also found a dose-dependent increases in BAX and cleaved Caspase-3 at the protein level (Figures 3E,F), which indicated that UA could enhance the apoptosis of endothelial cells.

### Overexpression of miR-214 Attenuated UA-Induced Apoptosis in MAECs

**Figure 4:**
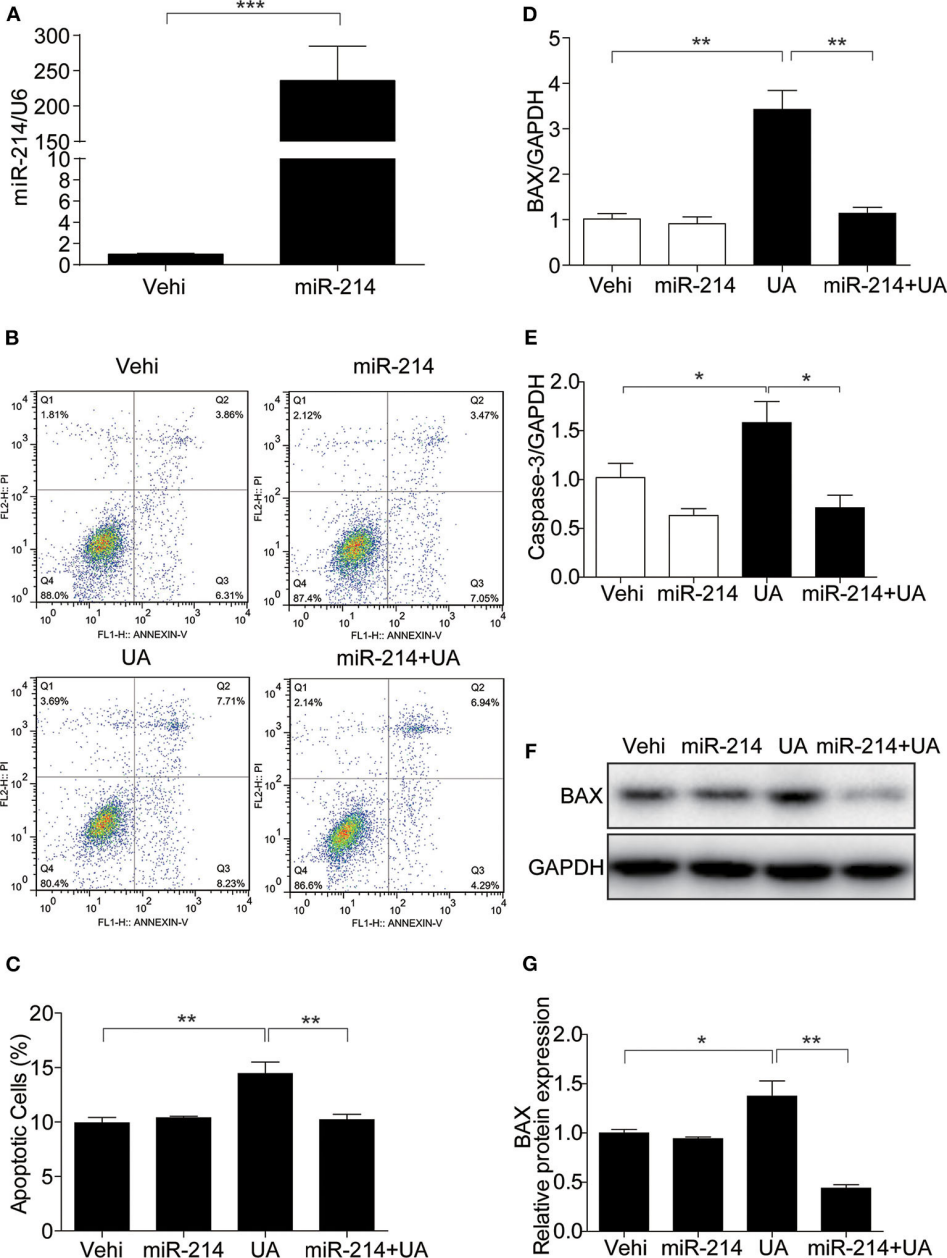
Overexpression of miR-214 attenuated UA-induced apoptosis in MAECs. To explore the potential role of miR-214 in UA-induced apoptosis, MAECs were transfected with miR-214 mimics or NC (negative control) for 24 h before UA (300 μM) administration. **(A)** The expression level of miR-214 was measured by qRT-PCR. **(B)** Apoptosis was assessed by FITC-Annexin V and PI staining, and representative images of the flow cytometry were obtained. **(C)** The quantification of apoptosis in **(B)**. **(D,E)** BAX and Caspase-3 mRNA expressions were analyzed by qRT-PCR. **(F)** Protein level of BAX was measured by Western blotting. **(G)** Densitometric analysis of BAX Western blots. GAPDH was used as an internal control. All values are means ± SE; *n* = 3 in each group. *P < 0.05, **P < 0.01, ***P < 0.001.

To investigate whether miR-214 regulates UA-induced apoptosis, the miR-214 mimic was transfected before UA administration (Figure 4A). The miR-214 mimic decreased the number of apoptotic cells (Figures 4B,C) and attenuated the UA-induced upregulation of BAX and Caspase-3 at the mRNA level (Figures 4D,E). In addition, the expression of BAX at the protein level was also blocked by the miR-214 mimic (Figures 4F,G). These results demonstrated that miR-214 could be of importance in attenuating UA-induced apoptosis in MAECs.

### COX-2 Was Upregulated in MAECs Following UA Stimulation

**Figure 5:**
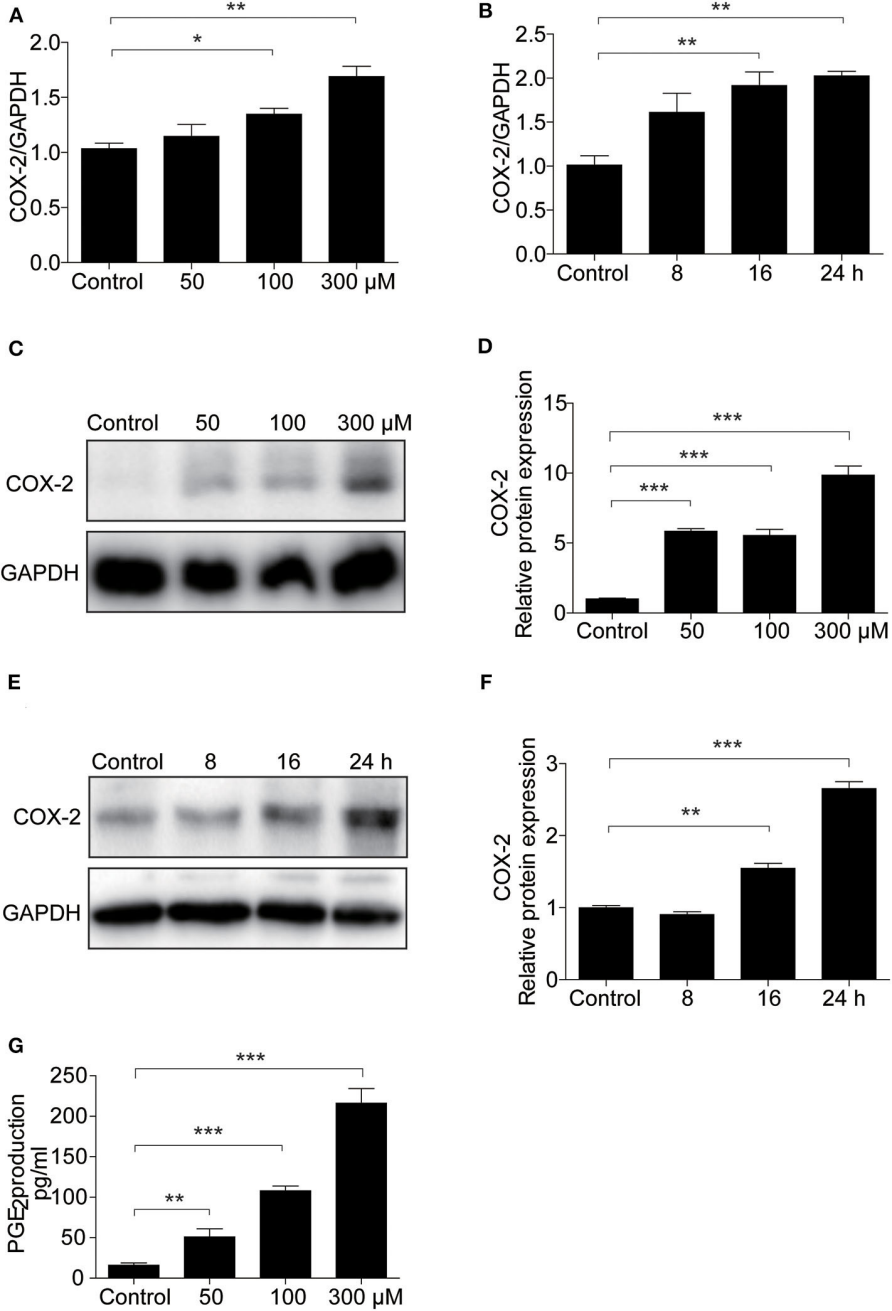
UA treatment increased COX-2 expression in MAECs. **(A)** qRT-PCR analysis of COX-2 mRNA expression in MAECs after treatment with different doses of UA (0–300 μM) for 24 h. **(B)** mRNA expression of COX-2 was determined in MAECs treated with UA at different time points. **(C)** Western blotting analysis of COX-2 in MAECs treated with different doses of UA (0–300 μM). **(D)** The densitometry of the blots in **(C)** was analyzed. **(E)** Time course analysis of COX-2 in MAECs treated with UA. **(F)** The quantification analysis of the Western blots in **(E)**. GAPDH was used as the loading control. **(G)** Enzyme immunoassay of PGE_2_ in the medium. All values are means ± SE; *n* = 3 in each group. *P < 0.05, **P < 0.01, ***P < 0.001.

To demonstrate the potential pathogenic mechanism involved in UA-induced apoptosis, COX-2 expression was assessed by Western blotting and qRT-PCR. We found UA clearly increased the mRNA level of COX-2 in a dose- and time-dependent manner (Figures 5A,B). Furthermore, the protein expression of COX-2 was increased by UA in a dose- and time-dependent manner (Figures 5C–F). In addition, the secretion of PGE_2_ was also significantly elevated after UA treatment (Figure 5G). These results indicated that COX-2 and PGE_2_ expression could be directly induced by UA in MAECs.

### Overexpression of miR-214 Blocked the UA-Induced Activation of COX-2 in MAECs

**Figure 6:**
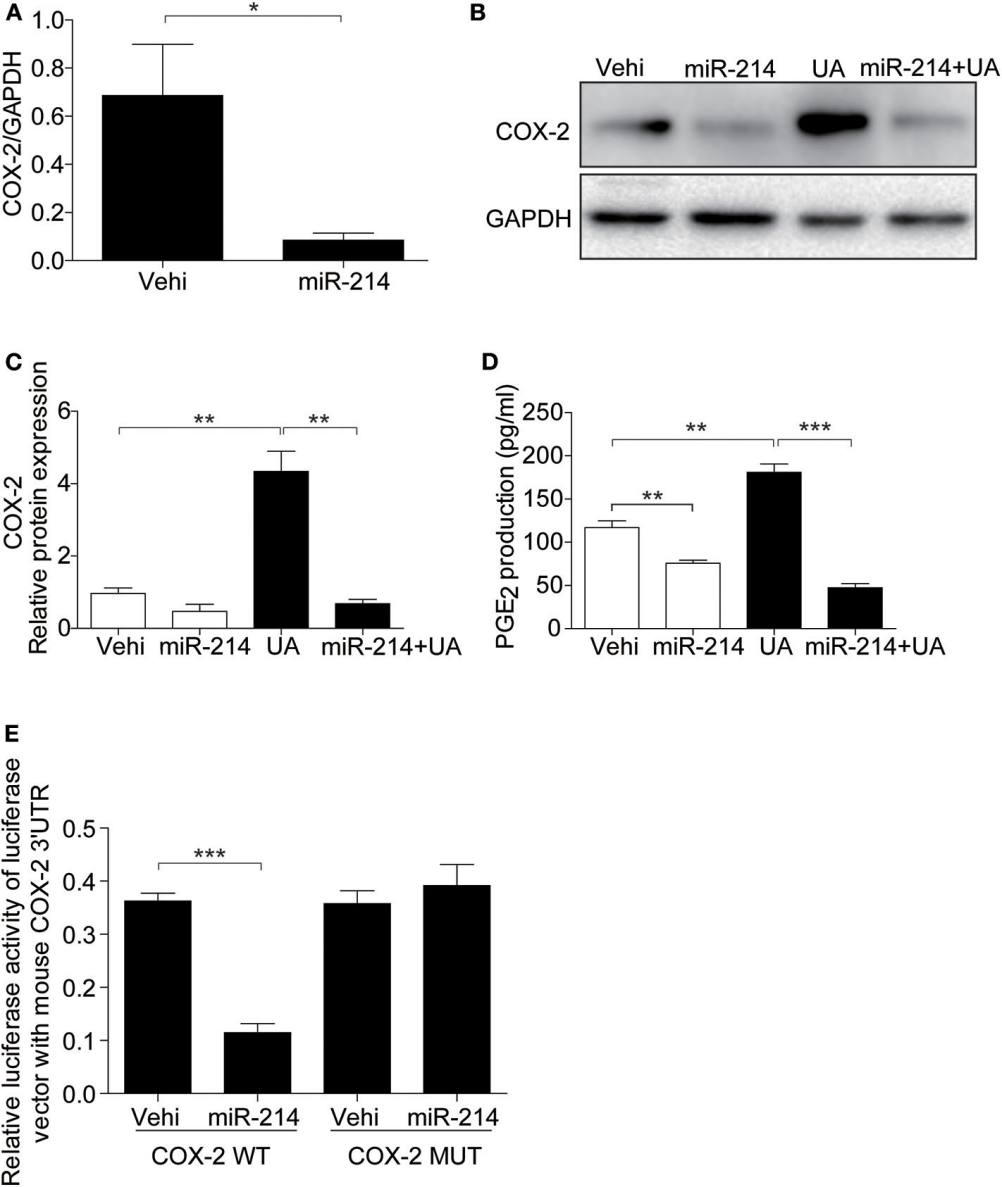
miR-214 directly targeted the 3′UTR region of COX-2 and inhibited its expression. The cells were transfected with miR-214 mimic for 24 h before UA (300 μM) administration. **(A)** qRT-PCR analysis of COX-2 mRNA expression after miR-214 mimic transfected. **(B)** Representative images of the Western blots of COX-2. **(C)** Densitometric analysis of the COX-2 in **(B)**. GAPDH was used as the loading control. **(D)** Enzyme immunoassay of PGE_2_ in the medium. **(E)** Luciferase reporter assay: MAECs were co-transfected with COX-2 luciferase reporter (WT) and miR-214 mimic or mutant COX-2 luciferase reporter (MUT) for 24 h. Transfected cells were lysed, and luciferase activity was measured. All values are means ± SE; *n* = 3 in each group. *P < 0.05, **P < 0.01, ***P < 0.001.

Furthermore, we examined the role of miR-214 in the UA-induced activation of the COX-2/PGE_2_ cascade. As expected, miR-214 overexpression inhibited the upregulation of COX-2 and the increase in PGE_2_ after UA treatment (Figures 6A–D). Luciferase activity assays further confirmed that miR-214 could directly target COX-2 in endothelial cells (Figure 6E). These data strongly suggest that the effect of miR-214 on alleviating UA-induced cell apoptosis could be mediated through regulating the COX-2/PGE_2_ cascade.

### Inhibiting COX-2 Abolished UA-Induced Apoptotic Response and PGE_2_ Production in MAECs

**Figure 7:**
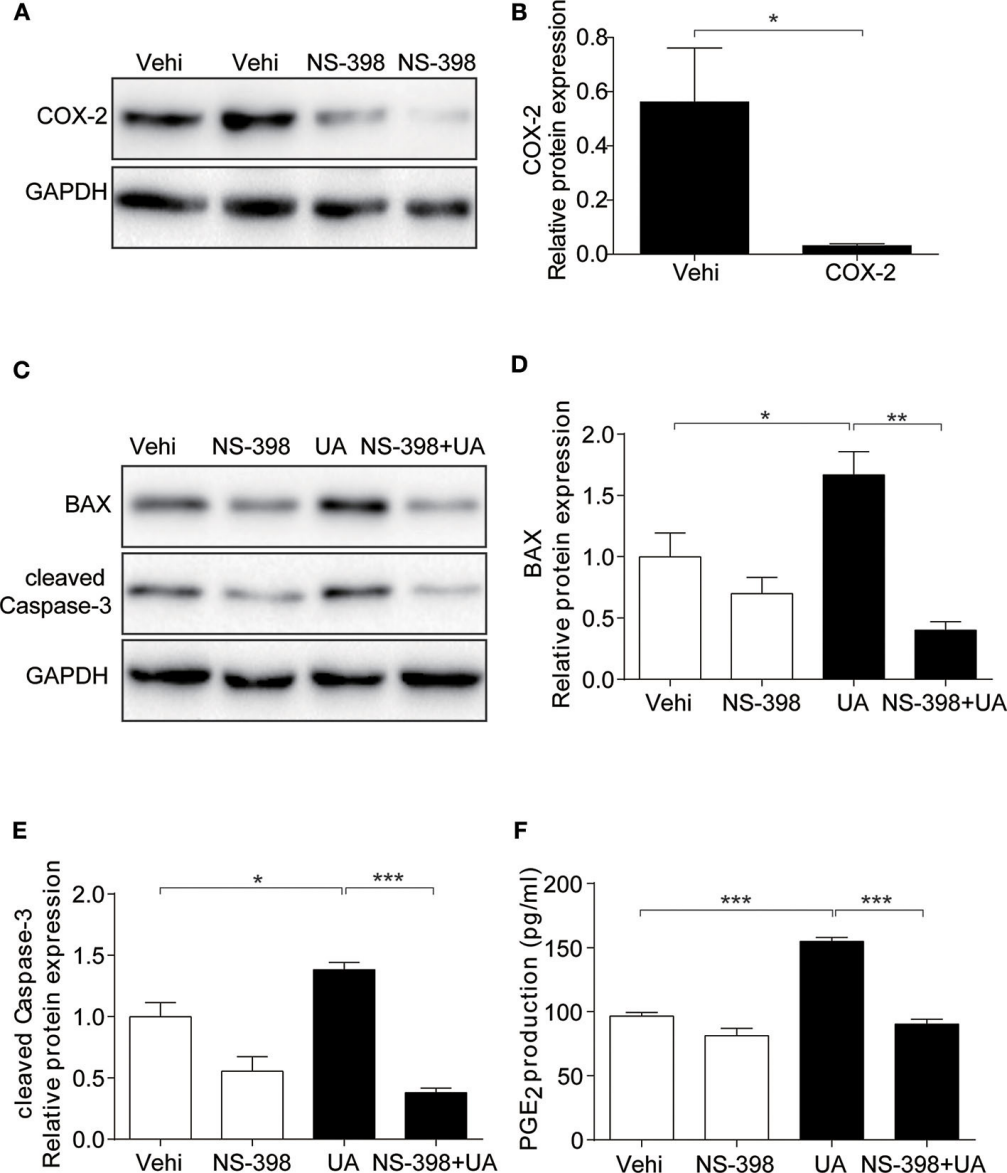
COX-2 inhibition abolished UA-induced apoptotic response in endothelial cells. The cells were pretreated with NS-398 (10 μM) for 12 h and then treated with UA (300 μM) for another 24 h. **(A)** Representative images of the Western blots of COX-2 expression after COX-2 inhibitor (NS-398) treatment. **(B)** Quantification of the Western blots of COX-2. **(C)** Protein levels of BAX and cleaved Caspase-3 were detected by Western blotting after the treatments of COX-2 inhibitor and UA in MAECs. **(D)** Quantitative analysis of the Western blots of BAX in **(C)**. **(E)** Densitometric analysis of the cleaved Caspase-3 in **(C)**. GAPDH was used as the loading control. **(F)** Enzyme immunoassay of PGE_2_ in the medium. All values are means ± SE; *n* = 3 in each group. *P < 0.05, **P < 0.01, ***P < 0.001.

As COX-2 was upregulated in MAECs treated with UA, we investigated whether apoptosis induced by UA was inhibited by the COX-2 inhibitor NS398. As shown in Figure 7, the COX-2 inhibitor decreased COX-2 expression at the protein level (Figures 7A,B). Meanwhile, inhibiting COX-2 markedly blocked the UA-induced upregulation of BAX and cleaved Caspase-3 at the protein levels (Figures 7C–E). Moreover, inhibition of COX-2 robustly abolished the induction of PGE_2_ following UA treatment (Figure 7F). These results suggest a critical role of COX-2/PGE_2_ in mediating UA-induced MAEC apoptosis.

## Discussion

Both clinical studies and basic research evidence suggest that hyperuricemia is associated with the development and progression of cardiovascular diseases (CVDs), while the pathogenic mechanisms remain elusive (26–28). A recent study demonstrated that a high blood level of UA was associated with vascular inflammation (29) and endothelial dysfunction, leading to CVDs (30). Another study reported that UA treatment of rats with middle cerebral artery occlusion enhanced vascular endothelial cell apoptosis (4). In our study, MAECs treated with different concentrations of UA showed increased expression of BAX and cleaved Caspase-3 and increases in the number of apoptotic cells, which confirmed the role of UA in promoting MAEC apoptosis as reported by a previous study (31). Furthermore, accumulating evidence has confirmed that miR-214 is a multifunctional miRNA in CVDs (32–35). However, the role of miR-214 in UA-induced endothelial cell apoptosis remains unknown.

miR-214 has been reported to be associated with CVDs and tumors but with elusive mechanisms (36–38). A recent study reported that miR-214 attenuated hepatocyte apoptosis by negatively regulating the TRAF1/ASK1/JNK pathway (39). Here, we observed decreased miR-214 in the circulation of hyperuricemia patients and MAECs treated with UA. Further analysis revealed a negative correlation between serum UA and circulating miR-214. In these hyperuricemia patients, they also showed relatively higher levels of BUN and serum triglyceride compared with healthy controls confirming an association between hyperuricemia and renal dysfunction and lipid disorders (40). Meanwhile, we also identified that COX-2 was upregulated in MAECs following UA treatment. Since miR-214 is downregulated in UA-induced MAECs, we transfected MAECs with a miR-214 mimic prior to UA treatment to observe its function. As expected, we found that the cell apoptosis, COX-2 expression and PGE_2_ production induced by UA were reduced by the miR-214 mimic, which suggested a role of miR-214 in regulating UA-induced apoptosis, possibly via suppressing the COX-2/PGE_2_ cascade.

To prove whether activation of the COX-2/PGE_2_ cascade contributes to the UA-induced apoptosis, we treated MAECs with a specific COX-2 inhibitor (NS398) before UA treatment. Obviously, the inhibition of COX-2 significantly alleviated the apoptosis caused by UA as indicated by the reduction in the number of apoptotic cells and the reductions in the expression of BAX and cleaved Caspase-3, which was consistent with the inhibition of PGE_2_ release. These results indicated a detrimental role of COX-2 in UA-induced endothelial cell apoptosis. Furthermore, using luciferase activity assays, we confirmed that miR-214 could directly target COX-2. COX-2 inhibitors have been widely used in the clinic for antagonizing inflammation. Thus, these results suggest that clinical application of specific COX-2 inhibitors such as celecoxib might be beneficial in preventing UA-associated cardiovascular diseases.

Moreover, we need to state that UA concentration around 300 μM was used to induce endothelial cell apoptosis. This concentration of 300 μM is definitely within the normal range of human UA levels. However, 500 μM UA caused very severe cell death in this cell line (data not shown). The reason could be explained by the difference between the *in vitro* and *in vivo* conditions. Unlike *in vivo* condition, this endothelial cell line was maintained in medium with 10% serum, thus, the cells might adapt to a low UA environment. For this cell line, 300 μM UA in medium already mimicked hyperuricemia and caused obvious cytotoxicity. In agreement with this concept, 0.3 mM (300 μM) UA triggered apoptotic response in human umbilical vein endothelial cells (HUVECs) (31).

In summary, we provide evidence that the dysregulation of miR-214/COX-2/PGE_2_ axis might serve as a new mechanism in mediating UA-induced endothelial cell injury. Enhancing miR-214 may ameliorate UA-induced endothelial injury by directly suppressing the COX-2/PGE_2_ cascade. Thus, miR-214 might be a potential target in the treatment of hyperuricemia-related endothelial injury and CVDs.

## Data Availability Statement

All datasets generated for this study are included in the article/supplementary material.

## Ethics Statement

The studies involving human participants were reviewed and approved by Human Subjects Committee of Nanjing Medical University. The patients/participants provided their written informed consent to participate in this study. Written informed consent was obtained from the individual(s) for the publication of any potentially identifiable images or data included in this article.

## Author Contributions

YZ, ZJ, and GD designed the experiment. BY, SL, and JZ performed the experiments and data analyses. BY and YZ drafted the manuscript. ZJ, YZ, GD, SH, and AZ revised and approved the manuscript. All authors contributed to the article and approved the submitted version.

## Conflict of Interest

The authors declare that the research was conducted in the absence of any commercial or financial relationships that could be construed as a potential conflict of interest.
